# Natural History of the Hymenoptera Venom Sensitivity Reactions in Adults: Study Design

**DOI:** 10.3390/ijerph19074319

**Published:** 2022-04-04

**Authors:** Simona Perčič, Lidija Bojanić, Mitja Košnik, Andreja Kukec

**Affiliations:** 1 National Institute of Public Health, Centre for Environmental Health, Zaloška 29, 1000 Ljubljana, Slovenia; andreja.kukec@mf.uni-lj.si; 2 The University Clinic of Pulmonary and Allergic Diseases Golnik, Golnik 36, 4204 Golnik, Slovenia; bojaniclidija7@gmail.com (L.B.); mitja.kosnik@klinika-golnik.si (M.K.); 3 Department of Internal Medicine, Faculty of Medicine, University of Ljubljana, Zaloška 7, 1000 Ljubljana, Slovenia; 4 Department of Public Health, Faculty of Medicine, University of Ljubljana, Zaloška 4, 1000 Ljubljana, Slovenia

**Keywords:** Hymenoptera venom allergy, risk factors, epidemiological association

## Abstract

Background: Allergic reactions to Hymenoptera stings can have varying levels of severity, according to the Müller grading system. Methods: By an epidemiological concept, this is a retrospective cohort study. The observed cohort was represented by patients referred to the University Clinic Golnik due to Hymenoptera allergic reaction in the period from 1997 to 2015. From the immunological database of the University Clinic Golnik, we obtained laboratory data (sIgE, skin tests and basophil activation test). The clinical characteristics of patients were obtained from BIRPIS. With the help of a questionnaire, which was sent to each patient in the period from May 2019 to April 2021, we obtained epidemiological data. For the assessment of the association between the severity of allergic reaction for the observed outcome, the severity of the first allergic reaction after Hymenoptera sting was used. Other variables were grouped according to risk factors. Discussion: We will identify the risk factors that could play an important role in a severe systemic reaction: the aetiology of the Hymenoptera sting, sex, age, history and severity of previous systemic reactions, being re-stung in an interval of two months, the frequency of re-stings, atopy, genetic predisposition, preventive medication use, other medication use, beekeeping or living next to beehives and why immunotherapy was not taken. Laboratory data will also be analysed to determine if there is any association with laboratory tests and the severity of the allergic reactions after Hymenoptera stings. Conclusions: Several new approaches are introduced in the study design. The most important is that the protocol covers epidemiological data gained from the questionnaire, as well as clinical data gained from the Immunological database and BIRPIS database. We expect to obtain significant results that will explain the risk factors for the natural history of Hymenoptera sting allergic reactions and will help allergologists, as well as general doctors, when facing those patients allergic to Hymenoptera venom without immunotherapy.

## 1. Introduction

In the previous five decades, interest in hypersensitivity reactions caused by stings of Hymenoptera (mainly bees (*Apis mellifera*), wasps (*Vespula vulgaris*) and hornets (*Vespa crabro*) living in colonies) has noticeably increased. Epidemiological studies of Hymenoptera stings in the general adult population show a sting prevalence of 56.6–94.5%, depending on the climate [1]. Allergic reactions to Hymenoptera stings can have varying levels of severity. Studies show that 0.3–7.5% of the general population reported having experienced systemic sting reactions (SSRs), and 2.4–26.4% reported having had large local reactions (LLRs) [2]. In 2011, an Austrian study was published that found that 3.3% of the general population have experienced SSRs, and 4.6% have experienced LLRs [3]. Since Slovenia borders Austria, and their cultural and climate conditions are very similar, we can expect similar results for Slovenia.

In most cases, sensitisation to Hymenoptera venom after a honeybee or wasp sting is clinically not relevant. Asymptomatic sensitisation (AS) to bee and wasp venom is frequently detected when measuring levels of specific IgE (sIgE) to Hymenoptera venom and is reported to be present in 27.2–40.7% of the general population [4,5]. Furthermore, AS is related to the total IgE (tIgE) levels; in healthy populations with high tIgE levels, sIgE to bee and wasp venom was demonstrable in up to 66.7% of the investigated individuals [5]. These facts are why the current criteria to diagnose Hymenoptera venom allergy cannot accurately predict the occurrence or severity of the SSR or even anaphylactic symptoms after a sting, either with bees or vespids.

Allergic reactions to Hymenoptera stings have varying levels of severity, which is not only true for the comparison between different individuals, as the stage of severity can also vary in each patient. According to the results of several studies, various risk factors may facilitate the development of Hymenoptera venom allergy and increase the severity of SSRs to Hymenoptera stings. A short interval between two stings is often followed by SSR to the second sting [6]. However, being stung very frequently appears to induce tolerance. This fact is most obviously seen in beekeepers. Those stung less than 10 times a year have a higher risk of SSR than those stung more than 200 times a year [7,8,9]. Severe SSR is associated with an elevated baseline serum tryptase [10]. In systemic mastocytosis, Hymenoptera sting reactions are most often severe [11]. Old age is another factor associated with the severity of SSR, and older patients with pre-existing cardiovascular and pulmonary disease, who take medications such beta-blockers or angiotensin-converting enzyme inhibitors, are at increased risk [12,13]. An atopic constitution may influence the symptoms of SSR and is associated with respiratory allergic reactions [14]. A study from Austria found that patients with Hymenoptera venom allergy and high levels of tIgE predominantly develop milder SSR, while patients with low tIgE more often develop severe SSR [15]. Other risk factors for SSR have been described as a consequence of exposure: (i) beekeepers and their family members or neighbours; (ii) work in a fruit or bakery shop or as a groundworker, gardener, firefighter or farmer; (iii) outdoor leisure activities like working in the garden, swimming, golfing or cycling and (iv) motorcycling [16].

The eventual goals of the epidemiological studies concerning Hymenoptera allergy are to elucidate the risk factors for severe/mild SSRs and the need for treatment with Hymenoptera venom immunotherapy to achieve a better quality of life or to prevent death by preventing anaphylaxis in affected patients. The current paper aims to describe a wide epidemiological approach for evidence-based new knowledge about the natural history of Hymenoptera venom sensitivity in adults.

## 2. Methods

### 2.1. Study Design and Study Population

This is a retrospective cohort epidemiological study design based on data from the Immunological database of the University Clinic of Respiratory and Allergic Diseases Golnik in Slovenia (University Clinic Golnik, Golnik, Slovenia), clinical data from the BIRPIS database of the University Clinic of Respiratory and Allergic Diseases Golnik in Slovenia and data collected by a questionnaire sent to patients from May 2019 to October 2020. For the study, we collected all data retrospectively: laboratory data (sIgE, skin test and basophil activation test (BAT)); clinical characteristics of the patients and data about the past characteristics after repeated stings (honeybee, wasp or hornet) from the time patients were examined in the hospital. The University Clinic Golnik immunological database covers patients referred due to SSRs and LLRs after Hymenoptera sting (bee, wasp and hornet) from 1997 to 2015. In our study, we included only patients without Hymenoptera venom immunotherapy. Patients were divided into two periods: those admitted to hospital from 1997 to 2004 and those admitted to hospital from 2005 to 2015, since the BAT (basophil activation test) was performed during the second period for some of them. There was no specific risk associated with participating in this study for the patients. The National Medical Ethics Committee of Slovenia approved this study (Academic research 0120-188/2017/4).

### 2.2. Study Area

The Republic of Slovenia is a nation-state in Southern Central Europe. It covers 20,273 square kilometres (7827 sq. mi) and had a population of 2,062,874 in 2015 [17]. There are four major climate systems: littoral Slovenia, Central Slovenia, the more continental part in North-eastern Slovenia and alpine Slovenia (the mountainous region). The territory of Slovenia is mainly hilly, with the exception of the Slovene littoral and the north-western area occupied by the Alps [18].

### 2.3. Recruiment of Patients

Before we started our research, the immunological database had to be prepared. The whole database, which contains 5657 patients admitted to the University Clinic Golnik, who were allergic to Hymenoptera sting (honeybee, wasp and hornet) during the two observed periods (those without Hymenoptera venom immunotherapy and those with Hymenoptera venom immunotherapy) was sent to the Republic of Slovenia Statistical Office in May 2019 to exclude patients who had died and to update the addresses of the participating patients. Excluded due to death were exactly 251 patients. One thousand, nine hundred and sixty-eight patients in the Immunological database were excluded from the study due to venom immunotherapy. Those patients with venom immunotherapy in our study were only unintentionally included in the recruitment, as the Immunological database is not perfect. We deliberately excluded those with Hymenoptera venom immunotherapy from our study, as we are currently interested only in the natural history of Hymenoptera venom sensitivity reactions. The allergic reactions after Hymenoptera stings for those with venom immunotherapy are expected to be different, and we intend to discover these kinds of allergic reactions in the following study. In our study, we included 3689 patients without immunotherapy. In the first period from 1997 to 2004, 1468 patients were included, and in the second period from 2005 to 2015, 2221 patients were included.

The first contact with the patients was a questionnaire about their history of bee, wasp or hornet stings sent to all included patients. In the envelope sent to the patient’s home address were (i) an invitation letter with a brief explanation of the study, (ii) a two-sided questionnaire and (iii) informed consent for agreement to participate in the study. Patients were asked to return the completed questionnaire and signed informed consent. From May to the end of June 2019, 2221 patients received the post. By the end of October 2019, 586 completed questionnaires with consent to participate were returned to us. In 56 cases, we received only the questionnaire without the signed informed consent. For these 56 patients, another invitation letter with informed consent was sent to be signed, and 18 patients returned the signed agreement. For the first period, the number of participants ready for analysis was 604. During March 2020, another 1430 envelopes were sent to the patients from the first period in the same manner as previously done. By the end of May 2020, 301 completed questionnaires with informed consent to participate were returned to us.

Since the response rate was not as high as we had expected, we decided to contact the patients again. We developed an internet questionnaire, which was identical to those sent previously to patients. To nonrespondents after the first round, we sent another invitation. It was a short letter inviting an individual patient to contact us through an e-mail specifically created for this study. After their contact, we promised to re-email them a link to the internet questionnaire. An informed consent form was included in the questionnaire. Nonrespondents aged more than 75 years were now excluded from the research. We also provided our telephone number for them to contact us for any explanation or information about the study. At the beginning of September 2020, 1350 invitation letters were sent to nonrespondents from the 2005–2015 period. After four weeks, we received 154 completed questionnaires. We received about 40 phone calls, of which 21 were from patients who did not have a computer but were interested in participating in the study. We sent them a questionnaire with an informed consent form to their home addresses, and 18 of them responded. For the first period from 1997–2004, the second round (invitation letter and internet questionnaire) was carried out in April 2021, when we sent 1003 invitation letters to nonrespondents. This time, we received 20 phone calls requesting information about the research. After three weeks, we received 72 completed questionnaires.

**Figure 1:**
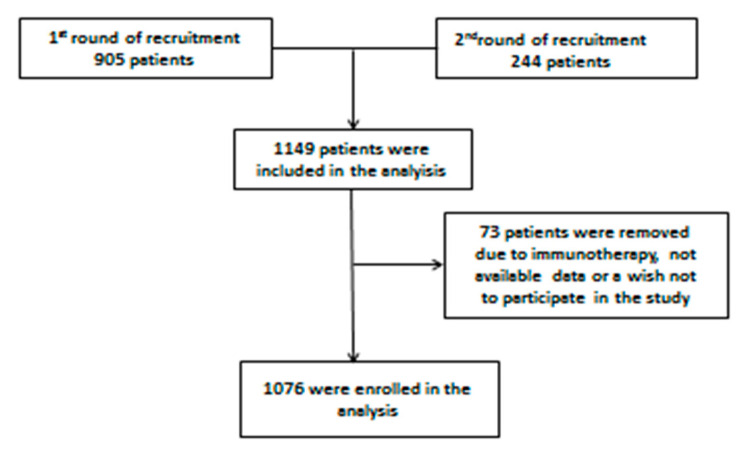
Flow chart for the final enrolment in the analysis.

We had to remove some of the respondents from our database. Some of the respondents were already treated with immunotherapy; for some, there were no available data in the BIRPIS database, and some of the respondents expressed a wish not to participate in the research. Due to these facts, we withdrew 73 of the respondent patients (Figure 1).

At the end of this process, 1076 patients were enrolled in theanalysis.

### 2.4. Data Collection

#### 2.4.1. Immunological Database

From the immunological database, we obtained a list of all patients admitted to University Clinic Golnik due to Hymenoptera venom allergy (honeybee, wasp or hornet) who did not have venom immunotherapy for the two observed periods; we obtained the age, sex, geographic position of the patient and aetiology of the sting. In this database, information on laboratory tests was also available. Each patient had at least one of the laboratory tests: sIgE for bee venom and/or wasp venom and/or hornet venom, skin test and BAT.

#### 2.4.2. BIRPIS Clinical Database

BIRPIS is a database covering all clinical data of the patients admitted to University Clinic Golnik. It also includes the history of each patient. For each patient, from the list of our participants, we examined the following characteristics: (i) aetiology of the first sting/stings, which was/were the cause for the allergy and admission to hospital, (ii) severity of the first allergic SSR or LLR (Mueller grading system), (iii) history of asthma, (iv) history of cardiovascular diseases, (v) atopic constitution (other allergies) and (vi) other medical therapy. When considering the assessment of the severity of the systemic allergic reactions at the Golnik Clinic, the Mueller grading system was used. The severity ranged from grade I to grade IV: grade I systemic reaction is characterised by generalised urticaria or erythema, itching, malaise or anxiety; grade II reactions may include symptoms associated with grade I reactions, as well as generalised oedema, tightness in the chest, wheezing, abdominal pain, nausea and vomiting and dizziness; grade III reactions may include symptoms associated with grade I or II reactions, as well as symptoms of dyspnoea, dysarthria, hoarseness, weakness, confusion and a feeling of impending doom and grade IV reactions may include symptoms associated with grade I, II or III reactions, as well as loss of consciousness, incontinence of urine or faeces or cyanosis. LLRs are characterised by oedema, erythema and pruritus, cover more than 10 cm in diameter and peak at between 24 and 48 h after the sting [19].

#### 2.4.3. Questionnaire

We created a questionnaire on the basis of published scientific literature (Appendix A: Qiestionnaire). There were 12 questions on the two-sided paper. We collected data from the recent past of the patients concerning Hymenoptera stings from the date they were admitted to hospital until the date of the sending them the questionnaire.

The questions were the following: (i) Have you ever had another sting (honeybee and/or wasp and/or hornet) after visiting our clinic, and how many of them? (ii) What were the signs of the allergic reaction (a table explaining the signs was included in the questionnaire)? (iii) Was the allergic reaction more severe, less severe or similar to the first allergic reaction? (iv) How long was the time from visiting our hospital to the next sting? (v) how many stings have you experienced since visiting the hospital? (vi) Do you have prescribed/take medicines/injection of adrenalin after you have been stung? (vii) Which medicine do you take? (viii) Does anybody in your family suffer from allergic reactions after bee and/or wasp and/or hornet stings? (ix) Do you live in an urban or rural area? (x) Are you a beekeeper, or do you live near a beehive? (xi) Do you often work in the garden? (xii) Why did you not treat your allergy with venom immunotherapy (if a doctor suggested it to you)?

The questionnaire was validated in terms of its content validity and face validity. Content validation aims to assess the relevance and representativeness of each item to a specific domain by the panel of experts. In this context, it will assess the relevance of all 15 items in the questionnaire to represent the usability domain. Content validation was conducted by 6 experts (3 public health experts and 3 allergologist experts) who were asked to give a score of 0 (item not relevant) or 1 (item very relevant). The Content Validity Index (CVI) was computed by calculating the scale average (Appendix A: Table A1). This was followed by face validation aims to assess the clarity and comprehensibility of the translated items. Twenty-four target users (15 with Hymenoptera venom allergy and 9 without) were selected for face validation. They were asked to give a score of 0 (item not clear and understandable) or 1 (item very clear and understandable) based on the clarity and comprehensibility of the questionnaire. The Face Validity Index (FVI) was computed by calculating the scale average (Appendix A: Table A2). The questionnaire was clear to the participants, and it took, on average, 13 min to answer all the questions. The Content Validity Index (CVI) (Appendix A: Table A1) and Face Validity Index (FVI) (Appendix A: Table A2) of the questionnaire were 0.966 and 0.963, respectively. A CVI and FVI of above 0.83 indicate that the items were relevant to the domain and clear and comprehensible for the use of target users [20].

### 2.5. Preparing the Data

All the data were prepared using Microsoft Excel, version 2010 (Microsoft, Redmond, WA, USA). Most data were converted into codes; those that were descriptive (alternative treatments the patients have used, allergies the patient has, other medication use and why immunotherapy was not used) remained uncoded. Finally, 46 variables were created. After all the data were prepared in Microsoft Excel, they were imported to IBM SPSS Statistic 27 and ready for statistical analysis.

### 2.6. Statistical Analysis

In the analysis, we used univariate and multivariate analytical approaches to elucidate the risk factors associated with an increased risk of a severe systemic allergic reaction after Hymenoptera sting.

In the analysis, we included the following variables:

Observed outcome: First Hymenoptera sting (bee, wasp or hornet) and the severity of the allergic reaction according to the Müller grading system.

Explanatory variables: History of repeated Hymenoptera sting and the severity of the allergic reaction according to the Müller grading system:

- skin prick test (noncontinuous variable; positive = 1, negative = 0).
- sIgE (continuous variable).
- BAT (continuous variable).

Other potential confounding factors:
Factors that increase the risk for repeated Hymenoptera stings:

- Sex (noncontinuous variable; male = 1, female = 0).
- Rural and urban area (noncontinuous variable; urban = 1, rural = 0).
- Beekeeping (noncontinuous variable; positive = 1, negative = 0).
- Farming (noncontinuous variable; positive = 1, negative = 0).

Factors that increase the risk for severity of repeated Hymenoptera stings:

- Age (noncontinuous variable (design of age groups: 1–20 years, 21–30 years, 31–40 years, 41–50 years, 51–60 years and 61–70 years).
- Genetic predisposition (history of Hymenoptera skin reaction in the family; noncontinuous variable; positive = 1, negative = 0).
- Cardiovascular diseases (noncontinuous variable; positive = 1, negative = 0).
- Atopic constitution (noncontinuous variable; positive = 1, negative = 0).
- Asthma (noncontinuous variable; positive = 1, negative = 0).
- The time from visiting our hospital to the next sting (noncontinuous variable; design of groups).
- The frequency of Hymenoptera stings (continuous variable).
- Preventive medication use ((1) antihistaminic and (2) corticosteroids; noncontinuous variable; only antihistaminic = 1, only corticosteroids = 2, both = 3 and none = 0).
- Injection of adrenalin (noncontinuous variable; not used = 0, used = 1).
- Use of other medications (noncontinuous variable; beta-blockers and angiotensin-converting enzyme inhibitors).

## 3. Discussion

To the best of our knowledge, this is the first extended retrospective study on the natural history of Hymenoptera venom sensitivity in adults. Previous studies on this topic were retrospective epidemiological observational studies [6,21,22,23,24,25,26,27], as well as prospective [28,29,30,31,32,33]. Most of these studies had a relatively small sample, except for the one by Björnsson and colleagues, in which the sample included 1815 patients [21]. No such extended number of variables were used and analysed in any of the individual studies. In our study, several important variables were examined. We attempted to explain the association between Hymenoptera (bee, wasp and hornet) venom allergy and the following characteristics:

### 3.1. Sensibilisation to Hymenoptera Venom

Bee and wasp venom sensitisation frequently occurs when measuring elevated levels of specific IgE (sIgE) to Hymenoptera venom and is reported to be 27.2–40.7% of the general population [4,15]. Many epidemiological studies emphasise that it is dependent on the rate of exposure to the Hymenoptera stings; for example, in Sweden, sensitisation is much lower than in other European countries because of the low exposure of the population [21]. However, beekeepers are more exposed, and the rate of sensitisation in the beekeeper population is much higher [9,23]. A higher prevalence of Hymenoptera venom allergy among men can be explained by higher exposure; men are exposed more because of active work outside or/and physically active outdoor exercise [21,27,30]. Additionally, the higher prevalence of sensitisation to wasps compared to bees in the general population is another factor affected by exposure. Wasps manifest aggressive behaviour and a marked tendency to share environments with people, because they are attracted by the foods and rubbish people produce. Bees, in contrast, have different eating habits, which interfere very little with people’s lifestyles. Their behaviour is much less aggressive unless they or their hives are disturbed [21]. Hornet nests are similar to wasp nests. They build their nests out of a pulp material primarily made from wood. Hornet nests are often found in eaves, trees, bushes and even underground. Hornets are among the most dangerous of stinging Hymenoptera insects, because they can sting repeatedly. Hornets are not as aggressive as some other types of wasps, but they can still be incredibly aggressive if they feel threatened [34].

### 3.2. Large Local Reaction

The prevalence of LLR varies widely in the populations studied and ranges from 2.4% to 26.4% [33]. In beekeepers, it is estimated to be 38.8% [9,23]. The causes of such a wide variation are not known; it has been suggested that the methodological aspects in the definition of the reaction or the exposure to Hymenoptera could be involved [24]. Patients with LLR tend to have the same reaction when re-stung. The risk of developing an SSR on being re-stung is low and varies from 5% to 10% [24]. Even though epidemiological studies indicate a clear difference in the natural histories between LLRs and SSRs, the standard methods of diagnosis (the skin test and sIgE level) are not able to discriminate between them.

### 3.3. Systemic Reactions

The prevalence of Hymenoptera SSRs in the general population has been the subject of various studies, which estimate it to be from 0.15% to 3.3% [21,33]. The degree of variability of these figures is mainly because of two influencing factors: the data collection technique and the degree of exposure to stings. The prevalence of SSRs in the general population in European countries is associated with the presence of Hymenoptera in the environment [21,33]. The higher prevalence of SSRs among beekeepers also confirms this hypothesis [9,23]. It is estimated that, after being re-stung, 17% of asymptomatic sensitised individuals react with SSRs [33]. In Austria, the most severe systemic reaction (anaphylactic reaction after Hymenoptera sting) is reported to be 17.5% of the total SSRs: however, 40% of insect sting fatalities occur in individuals with no history of a previous sting reaction [3].

### 3.4. Diagnostical Tests (Skin Test, tIgE, sIgE and BAT)

A total of 17% of the general population have been reported to have positive venom skin test responses [31]. The study confirmed that, in those allergic to wasp stings, with a positive history and a negative skin test, 22% have systemic reactions [32]; 10% of patients with negative skin tests or undetectable sIgE for insect venom react with anaphylaxis. However, it appears that, in a fairly large proportion (17%) of patients with a positive skin test and a negative history, the possibility of a systemic reaction still exists even more than 10 years after the evidence of a positive skin test [31]. Skin tests become negative in 30% of patients after two years and in almost 50% after three years [31]. From 27.1% to 40.7% of the population have positive sIgE. Given the prevalence of those who react with a systemic reaction, the test is not sensitive enough to predict a systemic allergic reaction. SIgE synthesis immediately after the sting is usually transient. The study also assessed the tIgE levels and their association with the systemic response. TIgE levels increase with age but not statistically significantly. Higher levels of tIgE were compared with the severity of the systemic response, but no association was found [26]. Higher levels of tIgE can perhaps also be attributed to atopy. Several studies have shown that atopy is associated with an increased risk of a systemic reaction after an insect sting [32]. The BAT proved to be a helpful additional tool because of its higher sensitivity [35] and specificity, lower rate of double-positive results and it has a predictive value for the severity of the reaction [36,37]. The purpose was to find reliable serological markers to predict tolerance to Hymenoptera stings.

### 3.5. Aetiology of the Sting

As we have already stated, the prevalence of Hymenoptera sting is higher for wasps than honeybees in our country. In another study, it was demonstrated that patients sensitive to honeybees may react repetitively with grade III and IV reactions more often than those sensitive to wasps [30]. This is a consequence of the fact that a honeybee may inject more venom with one sting [19]. However, most honeybee-sensitive patients were beekeepers, their family members or neighbours. They are characterised by being stung more often. Being aware of the risk of being stung again and not being easily frightened by a honeybee sting, they report to their doctor less frequently, with only a minor systemic reaction [30].

### 3.6. Sex

Studies about sex differences when reacting with SSRs after Hymenoptera stings usually focus on the role of sex hormones. Androgen receptors have been demonstrated on human mast cells, and oestrogens have been shown to enhance IgE-dependent mast cell activation. Being female is a risk factor for other immediate-type hypersensitivity disorders [27]. However, the prevalence of SSRs is generally higher for men due to the exposure factors previously described [21].

### 3.7. Age

Adults are more likely to have re-sting SSRs than children are; this is partially explained by different immunological responses not fully elucidated yet [38]. Those aged between 20 and 45 more often have SSRs than elderly people do, which is related to exposure. However, older people (45+ years old) have more severe SSRs after being re-stung, which is related to comorbidity, especially the presence of cardiovascular diseases and medication (beta-blockers or ACE inhibitors) [6,21].

### 3.8. History of Severe Systemic Reaction

The most important factor for the recurrence of SSR is the severity of the previous one. The disease tends towards spontaneous self-limitation, and the tendency to relapse varies from 21% to 73%, but the more severe the symptoms of the first sting, the more likely the reaction is to recur [30,39]. In studies that assessed the comparison of a reaction after a sting challenge to that after a field sting, the emotional state may influence different mediator systems in a patient (releasing neuropeptides and catecholamines), mediating the clinical severity. In addition, the description of symptoms after a field sting is influenced by subjective interpretation, whereas an in-hospital sting challenge can be assessed in a more objective way [30]. These facts must be considered when analysing the data.

### 3.9. Time between First Well-Tolerated Sting to Re-Sting

A specific risk factor for the occurrence of the first SSR is a well-tolerated sting in the previous two months [6].

### 3.10. The Frequency of Stings

A large number of simultaneous stings (>50) in one year may sensitise a person and then be followed by single-sting anaphylaxis, which was indicated in a study of beekeepers in Finland by Anilla and colleagues. The risk of SSRs was greatest during the first years of beekeeping, suggesting that spontaneous desensitisation occurs later [9]. Furthermore, Bousquet and co-workers found that beekeepers stung more than 50 times in a season do not develop any allergic reaction [40].

### 3.11. Atopy

Atopy is a risk factor for severe SSRs in beekeepers [31]. Moreover, during work with beehives, beekeepers are probably exposed to bee venom also via the eye, nasal and respiratory mucous membranes. The pathogenesis of bee venom allergy in beekeepers can be explained by sensitisation to bee venom via mucous membranes. Atopic beekeepers develop respiratory allergies to inhaled bee body debris and possibly to venom more frequently than non-atopic beekeepers [9].

### 3.12. Preventive Medication Use (Injection of Adrenalin, Antihistaminics and Glucocorticoids)

In Slovenia, prescribing prophylactic antihistamines and glucocorticoids is an example of good practice to prevent serious SSR. If a patient has had an experience of an SSR range of III or IV, according to the Müller grading, in the past and he/she has not taken immunotherapy, an injection of adrenalin is prophylactically prescribed. We will assess the prevalence of taking antihistamines and glucocorticoids after Hymenoptera re-stings and the prevalence of carrying/using adrenalin for auto-injection.

### 3.13. Use of Other Medications

It is well-known that some medications worsen the allergic response after Hymenoptera stings. In particular, these include medications for cardiovascular diseases (beta-blockers and angiotensin-converting enzyme inhibitors) [12]. We will assess the association of these medications with the severity of SSR.

### 3.14. Beekeepers and Living Next to Beehives

Beekeepers and their family members are heavily exposed to honeybee stings and are thus at an especially high risk of becoming allergic; therefore, they are an interesting population for the study of the epidemiology of venom allergy. We will assess how they are affected and what the differences from the general population are.

### 3.15. Geographical Area in Slovenia and Refusing Immunotherapy

We will assess which regions in Slovenia are where patients more commonly refuse to take immunotherapy. This is partially connected with the distance to the University Clinic Golnik, job engagement, not having time for any other reasons, poverty and not trusting the results of immunotherapy treatment.

## 4. The Strengths and Limitations of the Study Design

The study protocol introduced several new approaches addressing the problem of identification of the risk factors associated with the severity of systemic allergic reactions after Hymenoptera stings. First, the protocol addresses multiple variables in one unique study, which is not usual for the retrospective studies already conducted in this field. It covers epidemiological data gained from the questionnaire, as well as clinical data gained from the Immunological database and BIRPIS database. Second, we analysed all Hymenoptera, not only allergies to individual insects such as honeybees, wasps and hornets. Third, the study population covered all Slovenian patients admitted to the hospital due to allergic reaction after Hymenoptera stings from 1997 to 2015, as the University Clinic Golnik is the only one in Slovenia that covers diagnostic procedures and the treatment of this kind of allergy. Fourth, until now, no retrospective study in this field has included so many patients in the analysis. Fifth, this study was a very long-lasting retrospective study, so we expected to gain significant results. Further research is called for to discover and explore the potential of those novel approaches.

As far as data collection is concerned, the most used tool is the questionnaire. This approach has some limits. The problem is that different patients subjectively answered the same question differently even though they had had similar SSRs after re-stings. Greater control over the questionnaire results can be achieved with the supervision of an allergologist and by measuring the skin test and/or sIgE to confirm patients’ histories and thereby yield a picture of the data that is close to reality. For these reasons, the database of individual histories (BIRPIS) was extremely important in our study, as we had access to everyone’s history concerning the characteristics of individual health status and of the first sting.

## 5. Conclusions

Despite increasing interest in the natural history of Hymenoptera venom sensitivity in adults and studies being done, many facts still must be elucidated. Some risk factors are already known (aetiology, sex, age, history of systemic reactions, recurrence of stings in an interval of two months, frequency of stings, atopy, genetic predisposition and systemic mastocytosis), which should be discussed in the general population in preventing allergic reactions following a wasp, bee or hornet sting. The several new approaches of this study design compared to the already published studies in this field are that this kind of study design may provide more prediction information for patients allergic to Hymenoptera venom and improve their quality of life. The information from this study could also be used to guide medical attention and to properly shape interventions for allergic patients to Hymenoptera venom. The information regarding the problem of early diagnosis, treatment and improvement of prognosis among Hymenoptera allergic patients is particularly useful, as there are still many gaps that must be elucidated in the natural history of Hymenoptera allergic reactions. Promoting the importance of health in the natural course of the disease and on the effects of immunotherapy based on the scientific findings will undoubtedly improve the quality of life of patients suffering from such problems.

## Data Availability

The datasets used and/or analysed during the current study are available from the corresponding author upon reasonable request.

## Appendix A. Qiestionnaire

Name and surname:____________________________________

(**Q1)** Date of birth:_____________

**(Q2)** In the year___________you were examined in the University Clinic Golnik due to allergic reaction after Hymenoptera sting: wasp / bee / hornet.

**(Q3)** If the doctor advised you treatment with venom immunotherapy, please answer why you did not decide for immunotherapy? _______________________________

**(Q4)** After the visit in our Clinic, did you experienced another sting:

- Bee yes no (if yes: how many times? …………………)
- Wasp yes no (if yes: how many times? …………………)
- Hornet yes no (if yes: how many times? …………………)

**(Q5)** If you experienced another sting after visiting our Clinic, what were the signs after being re-stung:

**(Q6)** What was the severity of the reaction after being re-stung in comparison with the first sting?

- milder
- the same
- more severe

**(Q7)** How much time passed from the examination in the University Clinic Golnik to the next sting?

__________________________________________________________________________

**(Q8)** How many re-stings have you experienced?

___________________________________________________________________________

**(Q9)** After being re-stung, you usually:

- take medicines for prophylaxis       **yes  no**
- take an injection of epinephrin     **yes  no**

**(Q10)** Which medicines for prophylaxis of allergy do you take?

__________________________________________________________________________

**(Q11)** Does anybody in your familly suffer from Hymenoptera allergy (bee, wasp, hornet) (parents, brothers, sisters, children) ?

__________________________________________________________________________

**(Q12)** Do you live in urban or rural area?

__________________________________________________________________________

**(Q13)** Are you a beekeeper or you live next to the beehive?

__________________________________________________________________________

**(Q14)** Are you engaged in farm work?

__________________________________________________________________________

**(Q15)** Do you want to give us any another information?______________________________

**Table A1:** Content Validity Index based on the rating of the relavance of items by six experts.

	Expert1	Expert2	Expert3	Expert4	Expert5	Expert6	Expert in Agreement	L-CVI
Item								
**Q1**	1	1	1	1	1	1	6	1
**Q2**	1	1	1	1	1	1	6	1
**Q3**	1	1	1	1	1	1	6	1
**Q4**	1	1	1	1	1	1	6	1
**Q5**	1	1	1	1	1	1	6	1
**Q6**	1	1	1	1	1	1	6	1
**Q7**	1	0	1	1	1	1	5	0.8
**Q8**	1	1	1	0	1	1	5	0.8
**Q9**	1	1	1	1	1	1	6	1
**Q10**	1	1	0	1	1	1	5	0.8
**Q11**	1	1	1	1	1	1	6	1
**Q12**	1	1	1	1	1	1	6	1
**Q13**	1	1	1	1	1	1	6	1
**Q14**	1	1	1	1	1	1	6	1
**Q15**	1	1	1	1	1	1	6	1
							**CVI/average**	**0.966667**

**Table A2:** Face Validity Index based on the rating of the clarity and comprehensibility of items by 24 target users.

Item	RATER1	RATER2	RATER3	RATER4	RATER5	RATER6	RATER7	RATER8	RATER9	RATER10	RATER11	RATER12	RATER13	RATER14	RATER15	RATER16	RATER17	RATER18	RATER19	RATER20	RATER21	RATER22	RATER23	RATER24	Expert in Agreement	L-FVI
**Q1**	1	1	1	1	1	1	1	1	1	1	1	1	1	1	1	1	1	1	1	1	1	1	1	1	24	1
**Q2**	1	1	1	1	1	1	1	1	1	1	1	1	1	1	1	1	1	1	1	1	1	1	1	1	24	1
**Q3**	0	1	1	1	1	1	1	1	1	1	1	1	1	1	1	1	1	1	1	1	0	1	1	1	22	0.916667
**Q4**	1	1	1	1	1	1	1	1	1	1	1	1	1	1	1	1	1	1	1	1	0	1	1	1	23	0.958333
**Q5**	1	1	1	1	1	1	1	1	1	1	1	1	1	1	1	1	1	1	1	1	0	1	1	1	23	0.958333
**Q6**	1	0	1	1	1	1	1	1	1	1	1	1	1	1	1	1	1	1	1	1	0	1	1	1	22	0.916667
**Q7**	1	1	1	1	0	1	1	1	1	1	1	0	1	1	1	1	1	0	1	1	1	1	1	1	21	0.875
**Q8**	0	0	1	1	1	1	0	1	1	1	1	1	1	1	1	1	1	1	1	1	1	1	1	1	21	0.875
**Q9**	1	1	1	1	1	1	1	1	1	1	1	1	1	1	1	1	1	1	1	1	1	1	1	1	24	1
**Q10**	0	1	1	1	1	1	1	1	1	1	1	1	1	1	1	1	1	1	1	1	1	1	1	1	23	0.958333
**Q11**	1	1	1	1	1	1	1	1	1	1	1	1	1	1	1	1	1	1	1	1	1	1	1	1	24	1
**Q12**	1	1	1	1	1	1	1	1	1	1	1	1	1	1	1	1	1	1	1	1	1	1	1	1	24	1
**Q13**	1	1	1	1	1	1	1	1	1	1	1	1	1	1	1	1	1	1	1	1	1	1	1	1	24	1
**Q14**	1	1	1	1	1	1	1	1	1	1	1	1	1	1	1	1	1	1	1	1	1	1	1	1	24	1
**Q15**	1	1	1	1	1	1	1	1	1	1	1	1	1	1	1	1	1	1	1	1	1	1	1	1	24	1
																										**0.963889**
